# Persistent hypoglycemia in patients with liver cancer

**DOI:** 10.1530/EDM-23-0077

**Published:** 2024-07-02

**Authors:** Kemal Fariz Kalista, Hanum Citra Nur Rahma, Dicky Levenus Tahapary, Saut Horas Nababan, Chynthia Olivia Maurine Jasirwan, Juferdy Kurniawan, Cosmas Rinaldi Adithya Lesmana, Andri Sanityoso Sulaiman, Irsan Hasan, Rino Gani

**Affiliations:** 1 Division of Hepatobiliary, Department of Internal Medicine, Dr. Cipto Mangunkusumo National General Hospital, Faculty of Medicine Universitas Indonesia, Jakarta, Indonesia; 2 Department of Internal Medicine, Dr. Cipto Mangunkusumo National General Hospital, Faculty of Medicine Universitas Indonesia, Jakarta, Indonesia; 3 Division of Endocrinology, Metabolism and Diabetes, Department of Internal Medicine, Dr. Cipto Mangunkusumo National General Hospital, Faculty of Medicine Universitas Indonesia, Jakarta, Indonesia

**Keywords:** Adult, Female, Male, Asian - other, Indonesia, Liver, Endocrine-related cancer, Hyperglycaemia, Insight into disease pathogenesis or mechanism of therapy, July, 2024

## Abstract

**Summary:**

Hypoglycemia is one of the paraneoplastic syndrome manifestations that arise from primary and secondary liver cancer. Hypoglycemia usually presents in the late stage of the disease and indicates a poor prognosis. This case series displays the characteristics profile of patients with primary and secondary liver cancer who are presented with hypoglycemia in a tertiary referral hospital in Indonesia. The study included 41 liver cancer patients who were presented with hypoglycemia. Hepatocellular carcinoma was diagnosed in 51.2% of patients, metastatic liver disease in 14.6% of patients, and undiagnosed liver cancer in 34.1% of patients. The mean age was 47.7 years with male predominance (65.9%). Jaundice was found in 58.5% and hepatomegaly in 70.7% of patients. The mean (± S.D.) initial blood glucose was 42.15 ± 17.11 mg/dL and the Child–Pugh score was 9.93 ± 2.11. Based on imaging, tumor diameter was 12.6 ± 6.9 cm, multiple (61%), and involving both lobes (61%). Treatments for hypoglycemia included oral/enteral feeding, intravenous dextrose, and steroids. No treatment was given for the cancer because all patients were in an advanced stage. The treatment resulted in 41.5% blood glucose being controlled, 56.1% refractory, and 2.4% persistent. Mortality was 70.7% and in average occurred 5.76 ± 4.99 days after hypoglycemia. The mainstay of treatment in these cases is treating the tumor with cytoreduction. However, it was difficult to do cytoreduction because the tumor was already in an advanced stage. Beneficial supportive treatments for maintaining normal blood glucose are frequent meals, dextrose infusion, steroids, and glucagon.

**Learning points:**

- Hypoglycemia in liver cancer occurs due to the failure of the liver to fulfill body glucose demand because the liver parenchyma has been largely replaced by the tumor, in addition to the high production of insulin growth factor (IGF).
- Hypoglycemia is often caused by islet cell and non-islet cell tumors, with a higher occurrence in non-islet cell tumors due to paraneoplastic syndrome and the high metabolic requirements of the tumor.
- The mainstay of NICTH treatment is treating the tumor with cytoreduction. However, in an advanced stage, cytoreduction therapy is often challenging to conduct. Beneficial supportive treatments for controlling blood glucose are frequent meals, dextrose infusion, and the injection of steroids and glucagon.
- Steroids play a beneficial role in the treatment of persistent hypoglycemia in hepatocellular carcinoma by stimulating gluconeogenesis and increasing lipolysis. Steroids also have roles in the inhibition of peripheral glucose intake, suppression of big IGF-2 production, and modulation of the GH–IGF axis.

## Background

Hypoglycemia is an emergency condition that can be fatal if not detected early and treated immediately. Although most cases of hypoglycemia occur due to diabetes-mellitus-related treatment, hypoglycemia occurrence can also be caused by islet cell and non-islet cell tumors. Patients with non-islet cell tumors have a higher chance of suffering from hypoglycemia due to paraneoplastic syndrome and the high metabolic requirements of the tumor (1). Hepatocellular carcinoma (HCC) is the second most common cause of non-islet cell tumor hypoglycemia (NICTH). In HCC cases, the prevalence of hypoglycemia varies from 4% to 27% (2). Hypoglycemia usually occurs in an advanced stage of the disease where it is attributed to the large size of the tumor. However, several studies reported hypoglycemia as an early presentation of hepatocellular carcinoma (3, 4, 5). Hypoglycemia in advanced hepatocellular carcinoma is associated with poor prognosis (6). The objective of this study is to review the characteristics profile of patients with liver cancer who presented with hypoglycemia.

## Case presentation

In this study, we found 41 patients with liver cancer who presented with hypoglycemia. Moreover, 18 patients showed capillary or vein blood glucose level test results below 50 mg/dL at the time of initial hospital admission from July 2017 until February 2021 in Dr Cipto Mangunkusumo National General Hospital, a tertiary referral hospital located in Jakarta, the capital city of Indonesia. We included all patients with liver cancer regardless of the type and etiology. Hypoglycemia was diagnosed based on capillary or vein blood glucose level test results that were below 70 mg/dL. We defined controlled hypoglycemia as a single hypoglycemia event that was successfully corrected after treatment. Meanwhile, persistent hypoglycemia is a prolonged hypoglycemia event despite continuous treatment. Refractory hypoglycemia is described as repetitive hypoglycemia that requires repetitive treatment (1).

Table 1 shows the characteristics of these patients. More than half (65.9%) of the patients were male with a mean age of 47.7 years. Only 4.8% of the patients had type 2 diabetes mellitus. We found that 70.7% of patients had hepatomegaly, 63.4% had ascites, and 58.5% had jaundice. Cirrhosis was found in 36.6% of patients. Hepatocellular carcinoma was confirmed in 51.2% of the patients, metastatic liver disease in 14.6% of the patients, and 34.1% of the patients had an undetermined type of liver cancer. There were no adrenal issues like adrenal metastasis and adrenal insufficiency in this series of patients. Mortality was 70.7% and on average occurred 5.76 ± 4.99 days after hypoglycemia.

**Table 1:** Characteristics of the subjects. Data are presented as *n* (%) or as mean ± s.d.

Characteristics	Values
*n*	41
Age (years)	47.7 ± 12.56
Sex, male	27 (65.9)
Mortality	29 (70.7)
14-day mortality	28 (68.3)
Mortality after hypoglycemia (days)	5.76 ± 4.99
T2DM	2 (4.8)
Physical Examination	
Jaundice	24 (58.5)
Hepatomegaly	29 (70.7)
Splenomegaly	5 (12.2)
Ascites	26 (63.4)
Edema	14 (34.1)
Cirrhosis	15 (36.6)
Type of liver cancer	
HCC	21 (51.2)
Metastasis	6 (14.6)
NHL	3 (7.3)
Breast	1 (2.4)
Colon	2 (4.8)
Undetermined	14 (34.1)
Liver nodules	10 (24.4)
Liver + pulmonary nodules	3 (7.3)
Liver nodules + breast tumor	1 (2.4)
Response to the treatment	
Controlled	17 (41.5)
Persistent	1 (2.4)
Refractory	23 (56.1)

HBsAg, hepatitis B surface antigen; HCC, hepatocellular carcinoma; HCV, hepatitis C virus; NHL, non-Hodgkin lymphoma; T2DM, type 2 diabetes mellitus.

## Investigation

### Laboratory investigation

The results of the laboratory parameters of the patients are shown in Table 2. The mean initial blood glucose was 42.15 ± 17.11 mg/dL. The median bilirubin level was 4.88 (1.59–15.41) mg/dL. Hepatitis B surface antigen (HBsAg) was reactive in 57.6% of patients, while only 4.9% of patients were reactive for anti-HCV. Child–Pugh score mean was 9.93 ± 2.11**.**The median creatinine level was 0.90 (0.50–1.80) mg/dL. From the data, we also found that only two patients had reached end-stage renal disease and depended on dialysis.

**Table 2:** Laboratory parameters. Data are presented as mean ± s.d., *n* (%) or as median (IQR).

Characteristics	Values
*n*	41
Hemoglobin, g/dL	10.7 ± 2.72
Platelet, 10^3^/µL	299.5 ± 193.67
PT, second	15.6 (13.4–17.9)
INR, second	1.40 (1.23–1.72)
AST, U/L	279 (173–475)
ALT, U/L	65 (36–115)
Total bilirubin, mg/dL	4.88 (1.59–15.41)
Direct bilirubin, mg/dL	3.65 (0.91–11.37)
Indirect bilirubin, mg/dL	1.47 (0.52–3.50)
Albumin, g/L	2.56 (2.22–3.13)
Creatinine, mg/dL	0.90 (0.50–1.80)
Initial blood glucose, mg/dL	42.15 ± 17.11
Sodium, meq/L	129 (125–134)
HBsAg (+)	24 (58.5)
Anti-HCV (+)	2 (4.9)
Child–Pugh score	9.93 ± 2.11

AST, aspartate aminotransferase; ALT, alanine aminotransferase; HBsAg, hepatitis B surface antigen; HCV, hepatitis C virus; INR, international normalized ratio; PT, prothrombin time.

### Radiologic characteristics

We included all radiology modalities, including ultrasonography, CT scan, and MRI. The results are summarised in Table 3. Based on various imaging modalities, the mean of the tumor diameter was 12.6 ± 6.9 cm. The majority of the patients had multiple tumors (61%) and had tumors that involved both lobes (61%).

**Table 3:** Radiologic characteristics of liver cancer. Data are presented as mean ± s.d. or as *n* (%).

Characteristics	Total	Controlled	Persistent	Refractory
*n*	41	17	1	23
Size, cm	12.6 ± 6.9	9.2 ± 6.8	7.7	16.7 ± 5.4
Number of lesions				
Solitary	3 (7.3)	1 (5.9)	1 (100)	1 (4.3)
Multiple	25 (61.0)	11 (64.7)	0 (0)	14 (60.9)
Diffuse	13 (31.7)	5 (29.4)	0 (0)	8 (34.8)
Location				
Right lobe	16 (39.0)	5 (29.4)	1 (100)	10 (43.5)
Left lobe	0 (0)	0 (0)	0 (0)	0 (0)
Both lobes	25 (61.0)	12 (70.6)	0 (0)	13 (56.5)
PV thrombus	8 (19.5)	1 (5.9)	0 (0)	7 (30.4)

PV, portal vein.

## Treatment

Hypoglycemia was treated with a standard treatment protocol, including oral/enteral feeding and intravenous glucose. Intravenous steroids were not routinely given. Only 26.8% of the patients were given steroids based on the consideration of the treating physician. Specific cancer treatment was not given because all the patients were in advanced stages.

## Outcome and follow-up

The hypoglycemia treatment resulted in 41.5% of blood glucose being controlled; 56.1% was refractory, and 2.4% was persistent (Table 4).

**Table 4:** Response to hypoglycemia treatment. Data are presented as mean ± s.d. or as *n* (%).

Treatment	Total	Controlled	Persistent	Refractory
Initial blood glucose, mg/dL	42.15 ± 17.11	51.6 ± 13.1	17.9	36.8 ± 16.7
Hypoglycemia treatment				
Feeding + Dx	30 (73.2)	15 (88.2)	0 (0)	15 (65.2)
Feeding + Dx + steroid	11 (26.8)	2 (11.8)	1 (100)	8 (34.8)

Dx, dextrose.

## Discussion

**Figure 1:**
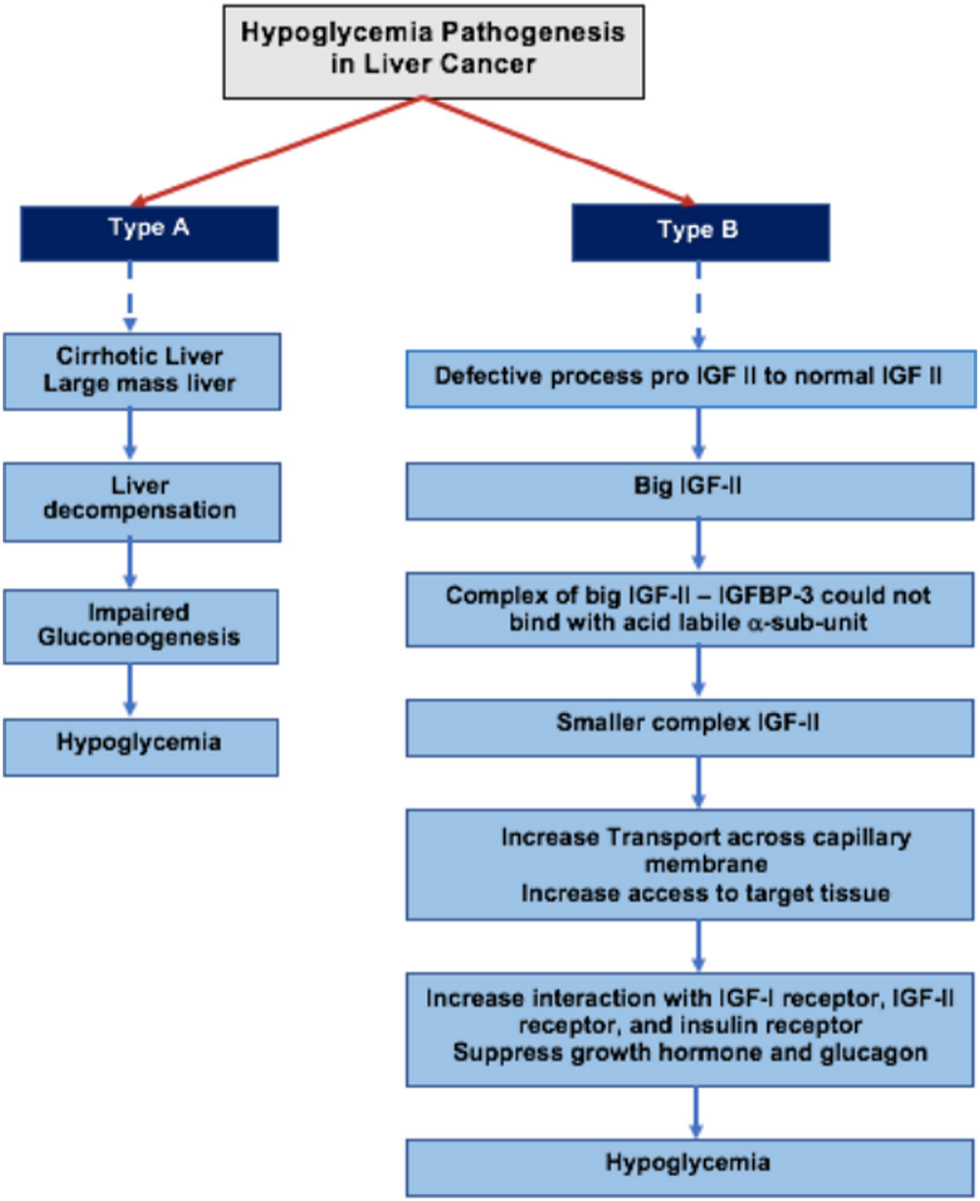
A flowchart for the understanding of the IGF–GH axis.

Hypoglycemia in liver cancer occurs due to the failure of the liver to fulfill body glucose demand because the liver parenchyma has been largely replaced by the tumor, in addition to the high production of insulin growth factor (IGF). Non-islet cell tumor hypoglycemia (NICTH) is divided into two types, i.e. type A and type B (7). Type A is often seen in advanced stages, when large-size tumors infiltrate liver parenchyma and consume high amounts of glucose in malnourished patients who already have depleted glycogen storage and defective gluconeogenesis (8). Type A NICTH usually occurs in late-stage disease when liver parenchymal damage is massive, such as in cirrhotic liver. Liver damage contributes to low levels of insulin and C-peptide, and escalating counterregulatory hormones (9). In type B NICTH, there is high production of big insulin-like growth factor II (IGF-II) by the tumors. Big IGF-II is formed by defective processing of pro IGF-II to normal-sized IGF-II (7.5 kDa). Normally, most of the IGFs are constructed in ternary complexes consisting of IGF, insulin-like growth factor binding protein-3 (IGFBP-3), and acid labile α-subunit. In this case, the big IGF-II–IGFBP-3 complex could not bind with the acid labile *α*-subunit, which resulted in a smaller complex that facilitates transport across the capillary membrane and enhances access to target tissues. Big IGF-II inhibits pituitary growth hormone (GH) secretion by binding the IGF-I receptor, which leads to decreased GH and IGFBP-3 (Fig. 1). This is usually found in the early stages of liver disease, which is characterized by overwhelming tissue glucose uptake with severe and persistent hypoglycemia (5, 8, 10, 11, 12, 13, 14).

To diagnose NICTH, analyze whether the condition of hypoglycemia fulfilling Whipple’s triad, measurement of serum insulin, C-peptide levels, counter-regulatory hormone, total IGF-II, big IGF-II, and 7.5 kDa IGF-II must be done to divine the type of NICTH (15). In Indonesia, we were unable to test the serum IGF-II level to ensure that hypoglycemia was caused by type B NICTH (10). In our study, most patients presented with jaundice and ascites, which indicated liver decompensation, high Child–Pugh score, and large-sized tumor, suggesting that type A NICTH was the cause of hypoglycemia. Additionally, our patients with refractory hypoglycemia had larger tumor sizes and lower initial blood glucose than patients with controlled hypoglycemia, hence increasing suspicion that type A NICTH was the cause of hypoglycemia. Moreover, although there were 18 patients with initial blood glucose below 50 mg/dL, there were no symptoms of losing consciousness, so the Whipple’s triad was not fulfilled.

In Nigerian patients, the median survival in hepatocellular carcinoma with paraneoplastic manifestation was 36 days. Patients with paraneoplastic syndrome resulting in erythrocytosis tended to have longer survival than similar patients with hypoglycemia and hypercalcemia. Child–Pugh’s score C, ineligibility for active treatment, serum alpha-fetoprotein level > 10 000 ng/mL, and main portal vein thrombosis indicate a poor prognosis (16). In our study, eight patients had portal vein thrombosis, and most of them showed refractory hypoglycemia after treatment was given. Portal vein thrombosis in hepatocellular carcinoma is associated with poor prognosis due to more aggressive tumor behavior, reduced tolerance to treatment due to portal hypertension, and deteriorated liver function reserve (17). Other conditions that can affect the blood glucose level are drug-induced hypoglycemia and liver cirrhosis. There were two patients with type 2 diabetes mellitus. Drug-induced hypoglycemia can be excluded as these patients were not given any hypoglycemic medication before or during hospital admission. In advanced cirrhotic liver, alteration of hepatic glucose metabolism had occured. The hepatic glucose output in response to glucagon is also decreased (6, 16).

Treatment of hypoglycemia in liver cancer is challenging. Patients with liver cancer are more likely to develop recurrent or refractory hypoglycemia. First, standard hypoglycemia treatment must be given. Besides correction of hypoglycemia, specific liver cancer treatment is needed to prevent recurrent hypoglycemia. The therapeutic options are cytoreduction with surgical resection and systemic chemotherapy. However, in advanced disease, cytoreduction is difficult to proceed with, and palliative treatments may be the best choice. In our cases, 17 patients had their hypoglycemia controlled by standard treatment, while one patient showed persistent hypoglycemia and 23 patients showed refractory hypoglycemia despite the provision of standard treatment. No specific treatment was given for the tumor because all the patients were in an advanced stage. The patients were given palliative treatment. In our cases, treatment for hypoglycemia was frequent feeding, dextrose infusion, and steroid injection. Huang and Chang reported that refractory hypoglycemia in advanced hepatocellular carcinoma could be controlled by systemic chemotherapy, and it should be considered when tumor removal surgery cannot be performed (6). Thipaporn *et al.* reported an effective and inexpensive method to treat persistent hypoglycemia in hepatocellular carcinoma, i.e. corticosteroid administration and frequent high carbohydrate intake. Steroids stimulate gluconeogenesis and increase lipolysis in adipocytes, while frequent high carbohydrate meals may fulfill the high demand for glucose, although sometimes it may not overcome glucose overutilization (14). In addition, steroids also have roles in the inhibition of peripheral glucose intake, suppression of big IGF-2 production, and modulation of the GH–IGF axis. High-dose glucocorticoid therapy, equivalent to prednisone 30–60 mg/day, has an immediate effect on hypoglycemia and is effective in correcting the underlying biochemical dysfunction (14, 18, 19, 20). Glucagon and GH, which are insulin counter-regulatory hormones, can be used as adjunctive (19, 21, 22). Glucagon releases glucose from liver storage to the bloodstream in the event of hypoglycemia. However, this activity decreases in patients with chronic liver disease; thus, continuous glucagon infusion might be favorable as a treatment for hypoglycemia caused by non-islet cell tumor (14, 23). Meanwhile, GH showed an effect in the stimulation of gluconeogenesis and glycogenolysis while increasing IGFBP-3 synthesis. This treatment modality should be used with caution due to the risk of stimulating tumor growth.

## Declaration of interest

The authors declare that there is no conflict of interest that could be perceived as prejudicing the impartiality of the study reported.

## Funding statement

This study did not receive any specific grant from any funding agency in the public, commercial, or not-for-profit sector.

## Patient consent

Written informed consent for the publication of the clinical details was obtained from the patients.

## Author contribution statement

The authors would like to express their sincerest gratitude to Dr Cipto Mangunkusumo National General Hospital, Faculty of Medicine, Universitas Indonesia for their continuous support. Kemal Fariz Kalista, Dicky Levenus Tahapary, Saut Horas Nababan, Chynthia Olivia Maurine Jasirwan, Juferdy Kurniawan, Cosmas Rinaldi Adithya Lesmana, Andri Sanityoso Sulaiman, Irsan Hasan, and Rino Gani are the physicians involved in the cases. Kemal Fariz Kalista and Hanum Citra Nur Rahma wrote the manuscript in consultation with Dicky Levenus Tahapary, Cosmas Rinaldi Adithya Lesmana, Irsan Hasan, and Rino Gani. Kemal Fariz Kalista, Dicky Levenus Tahapary, and Cosmas Rinaldi Adithya supervised the writing process of the manuscript. Kemal Fariz Kalista, Hanum Citra Nur Rahma, Dicky Levenus Tahapary, Saut Horas Nababan, Chynthia Olivia Maurine Jasirwan, Juferdy Kurniawan, Cosmas Rinaldi Adithya Lesmana, Andri Sanityoso Sulaiman, Irsan Hasan, and Rino Gani provided feedback and contributed to the final version of the manuscript.
